# Phosphatidylserine decarboxylase CT699, lysophospholipid acyltransferase CT775, and acyl-ACP synthase CT776 provide membrane lipid diversity to *Chlamydia trachomatis*

**DOI:** 10.1038/s41598-017-16116-8

**Published:** 2017-11-17

**Authors:** Eric Soupene, Frans A. Kuypers

**Affiliations:** Children’s Hospital Oakland Research Institute, Oakland, CA USA

## Abstract

*De novo* lipid synthesis and scavenging of fatty acids (FA) are processes essential for the formation of the membrane of the human pathogen *Chlamydia trachomatis* (*C*.*t*.). Host FA are assimilated via esterification by the bacterial acyl-acyl carrier protein (ACP) synthase AasC but inhibitors of the host acyl-CoA synthetase enymes ACSL also impaired growth of *C*.*t*. in human cells. In *E*. *coli*, activity of AasC was sensitive to triacsin C and rosiglitazone G. The absence of a triacsin C-insensitive pathway and the increased inhibition by rosiglitazone G confirmed the sensitivity of the bacterial acyl-ACP synthase to these drugs in infected human cells. We found no evidence that the human ACSL enzymes are required for lipid formation by *C*.*t*. The broad substrate specificity of acyltransferase CT775 provides *C*.*t*. with the capacity to incorporate straight-chain and bacterial specific branched-chain fatty acids. CT775 accepts both acyl-ACP and acyl-CoA as acyl donors and, 1- or 2-acyl isomers of lysophosphoplipids as acyl acceptors. The enzyme responsible for remodeling of human phosphatidylserine to bacterial phosphatidylethanolamine was identified as CT699. These findings provide evidence that the pathogen has the ability to extend the lipid diversity of its membrane.

## Introduction

The obligate intracellular bacterial human pathogen *Chlamydia trachomatis* (*C*.*t*.) has adapted its metabolic capacities to sustain its rapid growth in the confinement of the parasitophorous vacuole called the inclusion^1,2^. Lipids that are required to support its growth as well as to support the expanding size of the inclusion membrane are obtained by *de novo* lipogenesis and by scavenging of host fatty acids (FA) and lipids. Lipids of human origin, such as phosphatidylcholine (PC), phosphatidylinositol (PI), cholesterol, sphingolipids and ether lipids, are present in the bacterial membrane^3–6^. Several lines of evidence support the involvement of an intricate system of host and pathogen pathways in the acquisition and incorporation of these molecules. Several bacterial proteins are exported into the host compartment^7–9^, and various types of lipids, vesicles, lipid bodies, and proteins, as well as organelles with their enzymatic machineries, are translocated into the inclusion^3,10–16^. These entities are often detected adjacent to or in contact with the metabolically active reticulate form (RB) of the pathogen. Modifications of the proteome of the host cell, of lipid droplets (LDs) and of the infectious elementary form (EB) and RB of *C*.*t*., as well as the insertion of bacterial proteins into host vesicles in the inclusion and the exchange of straight-chain fatty acids for bacteria-specific odd-acyl chains, indicate that *C*.*t*. modifies the environment of its host to sustain its development^17–24^. The lack of a free-living culture system for *C*.*t*. and, until recently, the lack of tools for reverse genetic^25–27^ to study the metabolism of the dividing RB have hampered the understanding of these processes. The pathways that are used to modify imported host lipids by bacteria-specific acyl chains (odd-chain fatty acids^28,29^) are aspects of *C*.*t*. metabolism that are still controversial. The activity and function of *C*.*t*. enzymes that could participate in those pathways, such as the lysophospholipid acyltransferase CT775, have also been disputed^30,31^. In one study, remodeling of host phosphatidylserine (PS) head group to *C*.*t*. phosphatidylethanolamine (PE) was detected, but the bacterial PS-decarboxylase enzyme was not identified^5^.

In infected cells, incorporation of exogenous long-chain fatty acids into membrane glycerophospholipids proceeds by their esterification to acyl-CoA by human long-chain acyl-CoA synthetase (hACSL) enzymes and to acyl-ACP by the bacterial acyl carrier protein (ACP) synthase AasC (CT776)^6,32,33^. Both reactions require ATP. In addition, the cofactor co-enzyme A (CoASH) is also required since the ACP protein must be activated to holoACP. The formation of acyl-CoA by hACSL is inhibited by the drugs triacsin C and rosiglitazone G^32,34,35^. Treatment of infected cells with hACSL inhibitors results in impaired *C*.*t*. development, and the apparent immunodetection of hACSL proteins in the inclusion led investigators to propose an essential role for hACSL in lipid formation in the inclusion^36,37^. However, the effects of these drugs on the bacterial acyl-ACP synthase CT776 have not been assessed, and direct evidence establishing a role of host ACSL is still lacking. In *E*. *coli*, acyl-ACP formation is catalyzed by a bifunctional enzyme, Aas, that does not liberate acyl-ACP and is coupled to an acyl-ACP acyltransferase activity that transfers the acyl group to 2-acyl-GPL^38^. We wondered whether uncoupling of acyl-ACP formation by *C*.*t*. AasC would confer sensitivity of the bacterial enzyme to drugs that inhibit ACSL. To investigate this question, *in vivo* lipid labeling experiments and *in vitro* assessment of AasC activity were performed to determine the relative contribution of the acyl-CoA and acyl-ACP pathways to bacterial development when infected cells are exposed to these inhibitors. We established that CT775 is an acyltransferase with broad substrate specificity that can accept acyl-ACP and acyl-CoA as acyl donors and 1-acyl-GPL and 2-acyl-GPL as acyl acceptors. *In vivo* incorporation of 1-acyl-GPC in cells infected with *C*.*t*. confirmed the active remodeling of exogenous lipids that were translocated into the inclusions. Both the bacterial acyltransferase CT775 and human LPCAT1 could transfer odd-chain acyl-CoA (branched acyl-CoA) to 1-acyl-GPC to form PC, thereby providing evidence for the presence of a system in which host lipids are modified by the addition of bacterial branched FA within the inclusion. We identified the bacterial PS-decarboxylase enzyme that converts host PS to PE as CT699. These findings do not support the conclusion that members of the host ACSL family are required for lipid formation by *C*. *trachomatis* ^37^ and that modification of host lipids does not occur in the inclusion^6^.

## Results

### Fatty acid incorporation in Chlamydia-infected cells is inhibited by triacsin C

**Figure 1:**
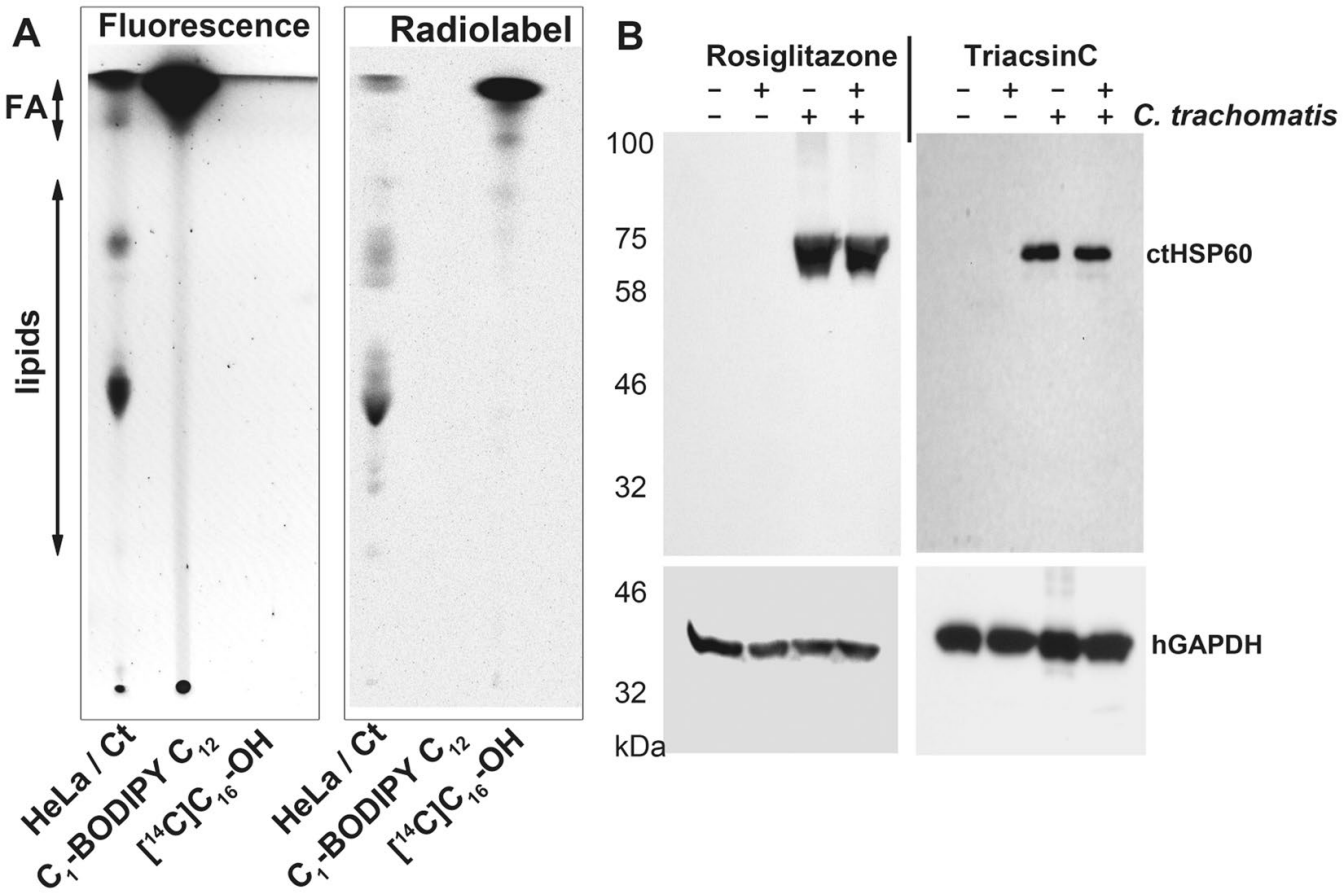
*In vivo* labeling of lipids in *Chlamydia*-infected cells. (**A**) HeLa cells were grown in T-75 flasks and were infected with *C*.*t*. or maintained uninfected. After 24 to 30 hours of infection, the cells were labeled by addition of 5 µM C_1_-BODIPY500/510 C_12_ to the medium, After incubation at 37 °C for an additional 2 hours, the cells were washed, and fresh medium containing triacsin C (10 µM), rosiglitazone G (100 µM) or ethanol (untreated cells) was added. After 1 hour of incubation at 37 °C, 10 µM ^14^C-C_16_-OH complexed with BSA was added to the medium. The cells were washed and harvested after a further 1-hour incubation at 37 °C (see Methods), and lipids and proteins were extracted. (**A**) Lipids were separated by thin-layer chromatography, and dually labeled lipids were visualized with a FluoChem camera and after exposure to a phosphorimager screen scanned with a StormImager. A sample obtained from HeLa-infected cells (HeLa/Ct) is shown in lane 1 of the TLC plate. C_1_-BODIPY500/510 C_12_ (lane 2) and ^14^C-C_16_-OH (lane 3) were used as migration standards. The migration positions of FA and phospholipids are indicated on the left. (**B**) Proteins were separated by SDS-PAGE, transferred to a PVDF membrane and blotted with monoclonal antibodies against *C*.*t*. HSP60 (upper panels) and human GAPDH (lower panel).

**Figure 2:**
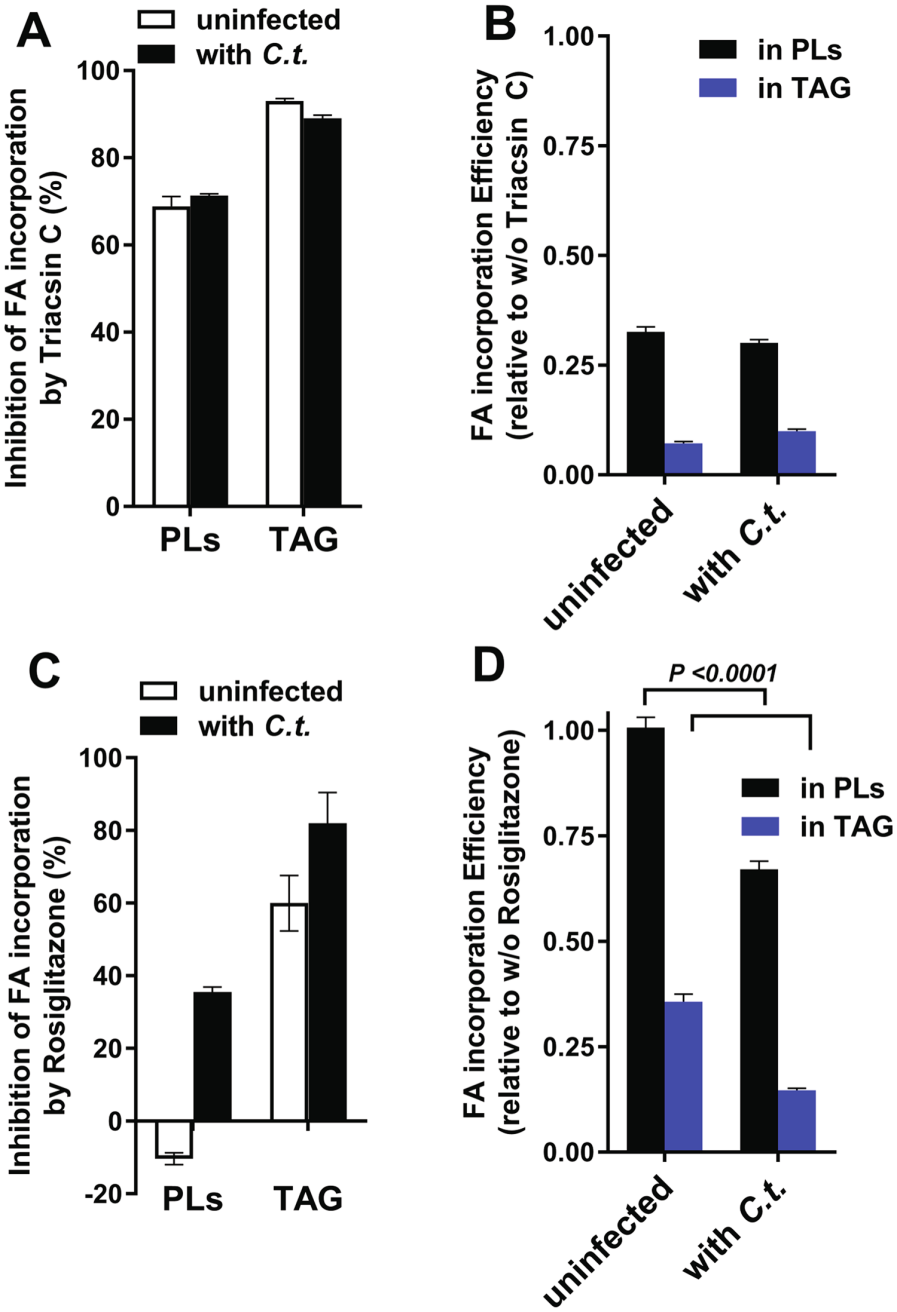
Quantification of *in vivo* FA incorporation in the presence of acyl-CoA synthetase inhibitors. Phospholipids and TAG obtained from dually-labeled HeLa cells infected with *C*.*t*. or maintained uninfected were separated by TLC, and fluorescence and radiolabeled lipids were quantified as described in the legend to Fig. 1. The cells were treated with 10 µM triacsin C and 100 µM rosiglitazone G, both of which were dissolved in ethanol. Ethanol was added to the untreated cells at the same final concentration as to the treated cells. The values obtained after labeling of lipids by the fatty acid analog C_1_-BODIPY500/510 C_12_ were used to calculate the % inhibition of incorporation of radiolabeled ^14^C-C_16_-OH (panels A and C). Values obtained with untreated cells were used to calculate the change in FA incorporation in the presence of the drugs (panels B and D). Note that whereas triacsin C inhibited FA incorporation in both uninfected and infected cells, rosiglitazone G did not reduce the incorporation of FA into glycerophospholipids of uninfected cells. The error bars indicate the standard deviation of 3 measurements of duplicated experiments.

The incorporation of FA into phospholipids (PL) was determined in uninfected cells before and after exposure of the cells to the drug triacin C for one hour. We reasoned that once the *in vivo* sensitivity of the acyl-CoA pathway in the host cells was established, the speculated but untested resistance of the bacterial acyl-ACP pathway to this drug could then be assessed in infected cells. The activity of a pathway that is not sensitive to inhibition would result in a higher rate of FA incorporation in infected cells than in uninfected cells in the presence of the drug. To further minimize the contribution of the sensitive acyl-CoA pathway and enhance the contribution of a resistant pathway for FA incorporation in infected cells, treatment was performed 24 hours post-infection when the *C*.*t*. inclusion occupied most of the cytosol of the cells. First, the cells were labeled with the fluorescent fatty acid analog C_1_-BODYPI-500/510-C_12_. The label was chased to deplete the unreacted pool of the compound, and drug (triacsin C or rosiglitazone G) was added to the medium. The cells were then incubated with [^14^C]C_16_-OH. Lipids were extracted and fluorescently labeled, and ^14^C-labeled PLs were separated and quantified (Fig. 1A). Proteins were also extracted and analyzed by immunodetection of bacterial HSP60 and human GAPDH proteins (Fig. 1B). The ratio of radiolabeled PLs to fluorescent PLs in the untreated and treated infected cells was compared to the ratio in uninfected cells (Fig. 2). Triacsin C was a potent inhibitor and approximately 70% reduction of PL formation was observed in both infected and uninfected cells after treatment (Fig. 2A). Strong inhibition of triacylglycerol (TAG) formation was also detected. The lack of resistance of FA assimilation pathways in infected cells to the drug (Fig. 2B) suggested that formation of acyl-ACP by the *C*.*t*. enzyme was sensitive to triacsin C.

### Fatty acid incorporation by Chlamydia-infected cells is inhibited by rosiglitazone G

Treatment of HeLa cells with *rosiglitazone G* (RG) did not result in inhibition of FA incorporation into PLs (Fig. 2C,D). Similar results have been observed in other cell types^34^. This lack of inhibition was confirmed by the observation that incorporation of the residual C_1_-BODYPI-500/510-C_12_ present in the cells, which represented the remaining portion of the label that was added to the medium prior to addition of the drug, further increased during the treatment period (negative values shown in Fig. 2C). Additionally, as established in other cell types^34^, RG treatment resulted in inhibition of TAG formation. Thus, the effect of RG on *C*.*t*. development is not the result of decreased activity of host enzymes that catalyze PL synthesis from acyl-CoA. However, in agreement with the finding of a bacterial developmental defect in RG-treated cells, a 60% reduction in PL labeling was detected in cells infected with *C*.*t*.in the presence of the drug. The TAG level was also lower in infected cells than in uninfected cells (Fig. 2C,D). In infected cells with large inclusions, the activity of a putative bacterial triacsin C and RG-resistant system should have been detected. In contrast, incorporation of exogenous FA was more sensitive to RG treatment in the presence of the bacteria. These data strongly suggest that the proposed essential function of the host ACSL enzymes in *C*.*t*. development, which was suggested solely based on the inhibitory effect of these drugs^37^, should be reconsidered.

### Chlamydia AasC activity is inhibited by triacsin C and rosiglitazone G

**Figure 3:**
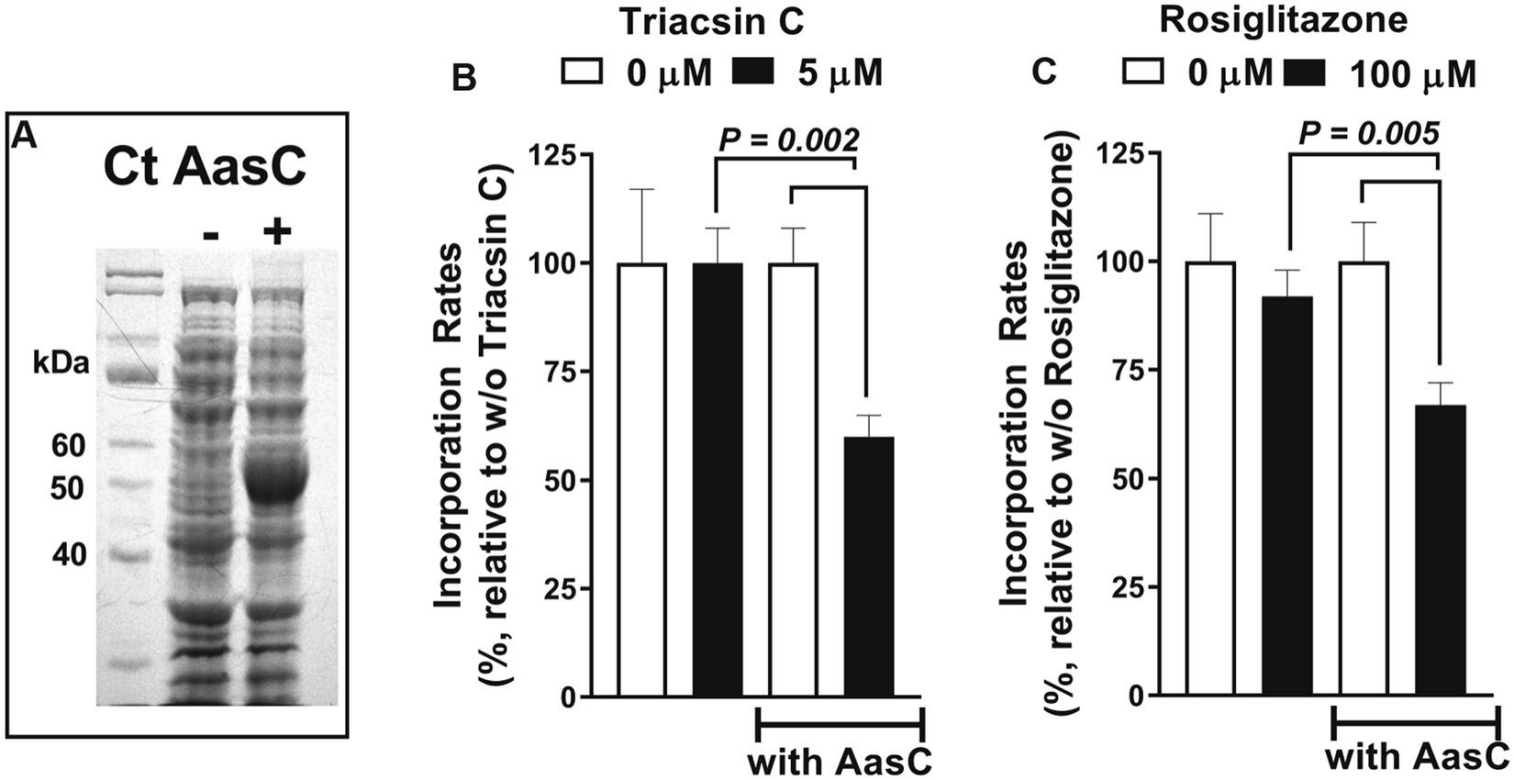
Sensitivity of the *C*.*t*. acyl-ACP synthase AasC to acyl-CoA synthetase inhibitors. AasC (CT776) was expressed in *E*. *coli*, and the incorporation of 5 µM ^14^C-C_16_-OH was determined in crude lysates in the presence of 5 µM triacsin C and 100 µM rosiglitazone G, as indicated. The reactions were performed at 30 °C; the rate of ^14^C-PL formation was calculated from measurements of 4 samples taken at intervals between 0 and 8 min. The values obtained in the absence of the drugs were used to calculate % inhibition in their presence. (**A**) *E*. *coli* proteins before and after induction of expression of *C*.*t*. AasC were separated by SDS-PAGE and stained with the GelCode Blue dye. Panels B and C. Quantification of the incorporation of ^14^C-C_16_-OH in the presence of triacsin C (panel B) and rosiglitazone G (panel C) is reported as percentage relative to the incorporation observed in their absence. Three independent experiments were performed and the error bars indicate the standard deviation of 3 measurements.

To address the *in vivo* inhibition of bacteria-driven FA incorporation by triacsin C and RG, the activity of the acyl-ACP synthase AasC (CT776) was measured in *E*. *coli*. AasC catalyzes the esterification of FA to acyl-ACP in the presence of holoACP^31,38^. AasC was efficiently produced in *E*. *coli* (Fig. 3A), and PE formation was analyzed in cell lysates in the presence of radiolabeled [^14^C]C_16_-OH (Figure S1). Control reactions confirmed that the activity of *E*. *coli* acyl-CoA synthase FadD was not required in lysed cells and that the reaction was ATP-dependent (Figure S1, panels A and B). The addition of lysoPC drove the reaction toward formation of PC (Figure S1C), establishing that the generated acyl-ACP was available for the acyltransferase reaction. The absence of PL formation from [^14^C]C_16_-CoA confirmed that incorporation of [^14^C]C_16_-OH was dependent on the acyl-ACP synthase activity (Figure S1D).

In the absence of AasC, [^14^C]C_16_-OH incorporation into *E*. *coli* lipids was not affected by triacsin C or rosiglitazone G (Fig. 3B,C). However, both drugs had a significant inhibitory effect on FA incorporation in the presence of the AasC enzyme, indicating that *Chlamydia* acyl-ACP synthase was a target of inhibition by these drugs *in vivo*. Thus, the inhibitory effects of these drugs on *C*.*t*. development in infected human cells cannot be simply attributed to the inhibition of the host ACSL enzymes, as has previously been suggested^37^.

### Chlamydia PS-decarboxylase CT699 converts PS to PE

**Figure 4:**
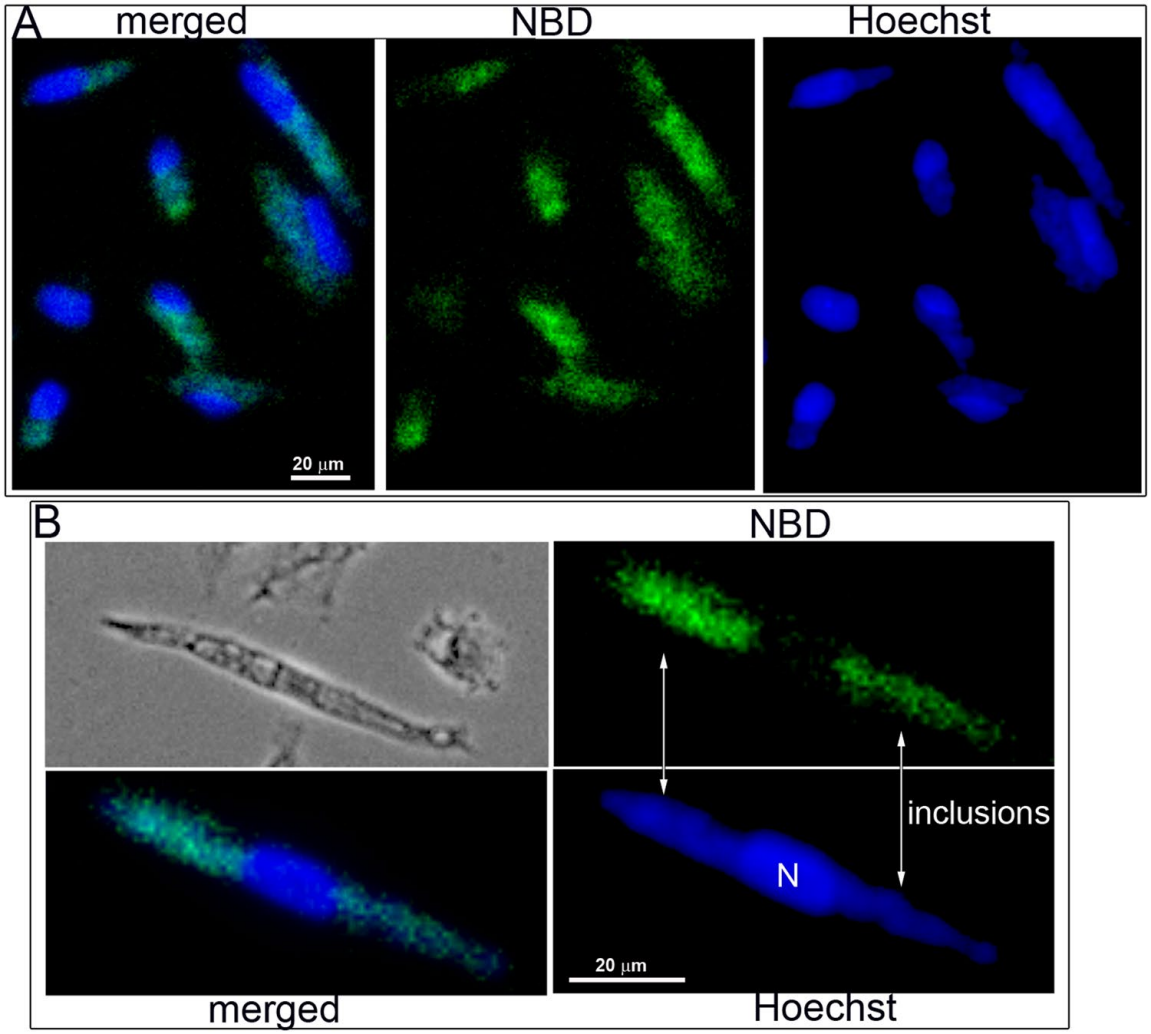
Labeling of *Chlamydia*-infected cells with NBD-PS. HeLa cells were grown on coverslips and infected with *C*. *trachomatis* strain D. After 24 hours, 1 µM NBD-PS (green) was added to the medium. The cells were washed and fixed after 1 hour of incubation. DNA was stained with Hoechst dye (blue), and imaging was performed with a Keyence microscope equipped with a 40x objective. Panel B shows a magnified cropped image of an infected cell with 2 inclusions, which are indicated. Images were taken from a single labeling experiment.

Labeling of the inclusion membrane with fluorescent phosphatidylserine (PS) molecules was reported by others^39^, and labeling of host PS resulted in the presence of labeled PE in the bacterial membrane^5^. We confirmed that addition of NBD-PS to the culture medium of human cells infected with *C*.*t*. resulted in intense labeling of the inclusions. Although the host cell contains a PS-decarboxylase (PISD) and a PS-synthase (PTDSS1) that can convert PS to PE^40^, fluorescent labeling was predominantly detected in the inclusions of the infected cells (Fig. 4).

**Figure 5:**
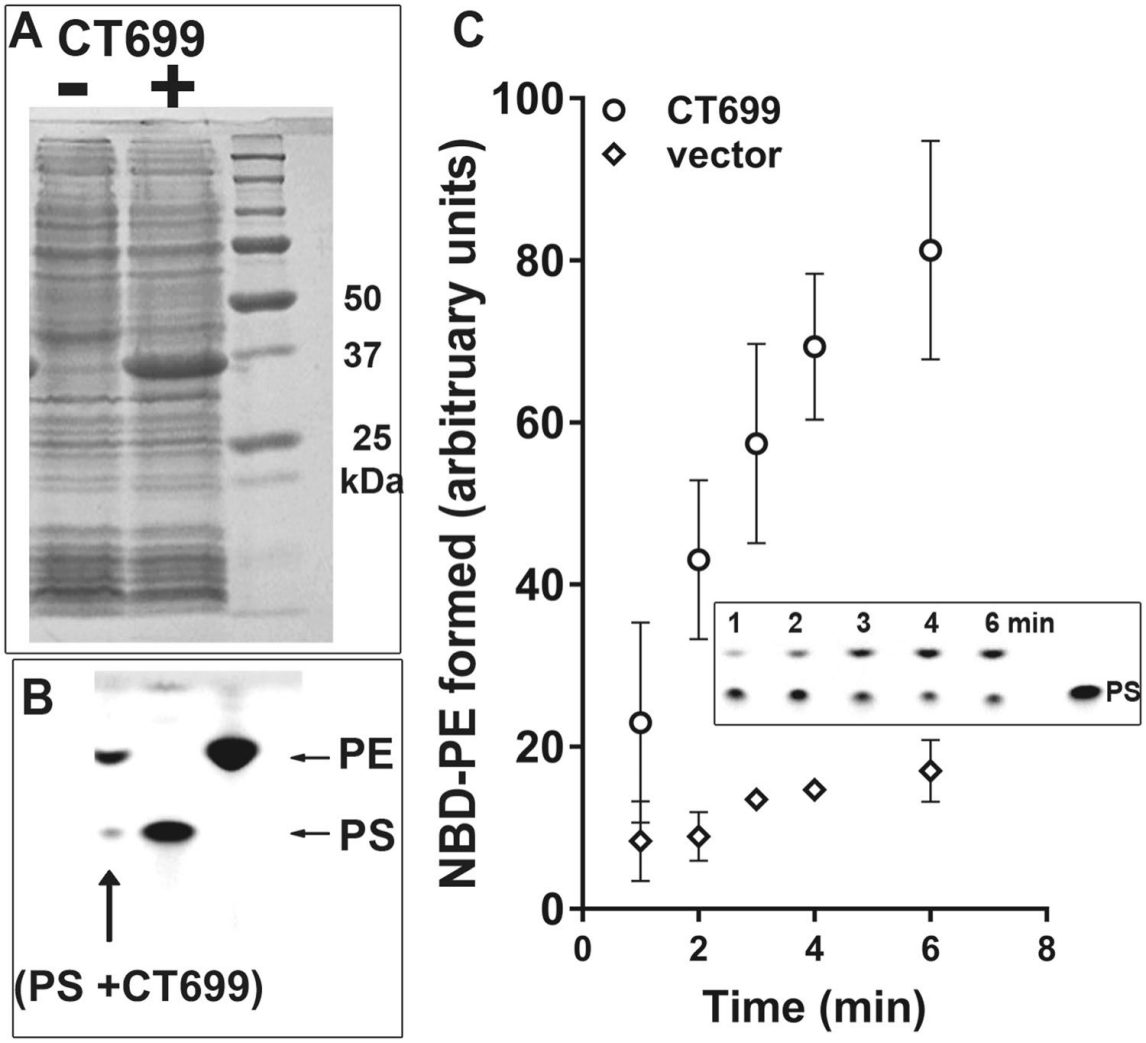
CT699 is a PS-decarboxylase enzyme. CT699 was expressed in *E*. *coli* (panel A), and activity measurements were performed in the presence of 2 µM NBD-PS at 37 °C for 20 min (panel B, lane PS + CT699) or from 0 to 6 minutes (panel C). Control reactions were performed using *E*. *coli* cells without the cloned CT699 construct (vector, panel C). Lipids were extracted and separated by thin-layer chromatography using unreacted NBD-PS and NBD-PE as migration standards (panel B and insert panel C). Several independent experiments were performed and the error bars in panel C indicate the standard deviation of 3 measurements of the samples obtained in one experiment.

The annotated *CT699* (*PsdD*) gene was predicted to encode a PS-decarboxylase enzyme. To test its function, the protein was produced in *E*. *coli*. CT699 was active and able to convert fluorescent NBD-PS into NBD-PE (Fig. 5A,B). Activity of ecPsdD was also detected under those conditions, but PS decarboxylation was significantly greater in the presence of CT699 (Fig. 4C). Together, these data establish that host PS molecules can be taken up by the inclusion and that conversion to PE is catalyzed by *Chlamydia*.

### Fate of 1-acyl-sn-glycerol-3-P-choline (1-acyl-GPC) in Chlamydia-infected cells

**Figure 6:**
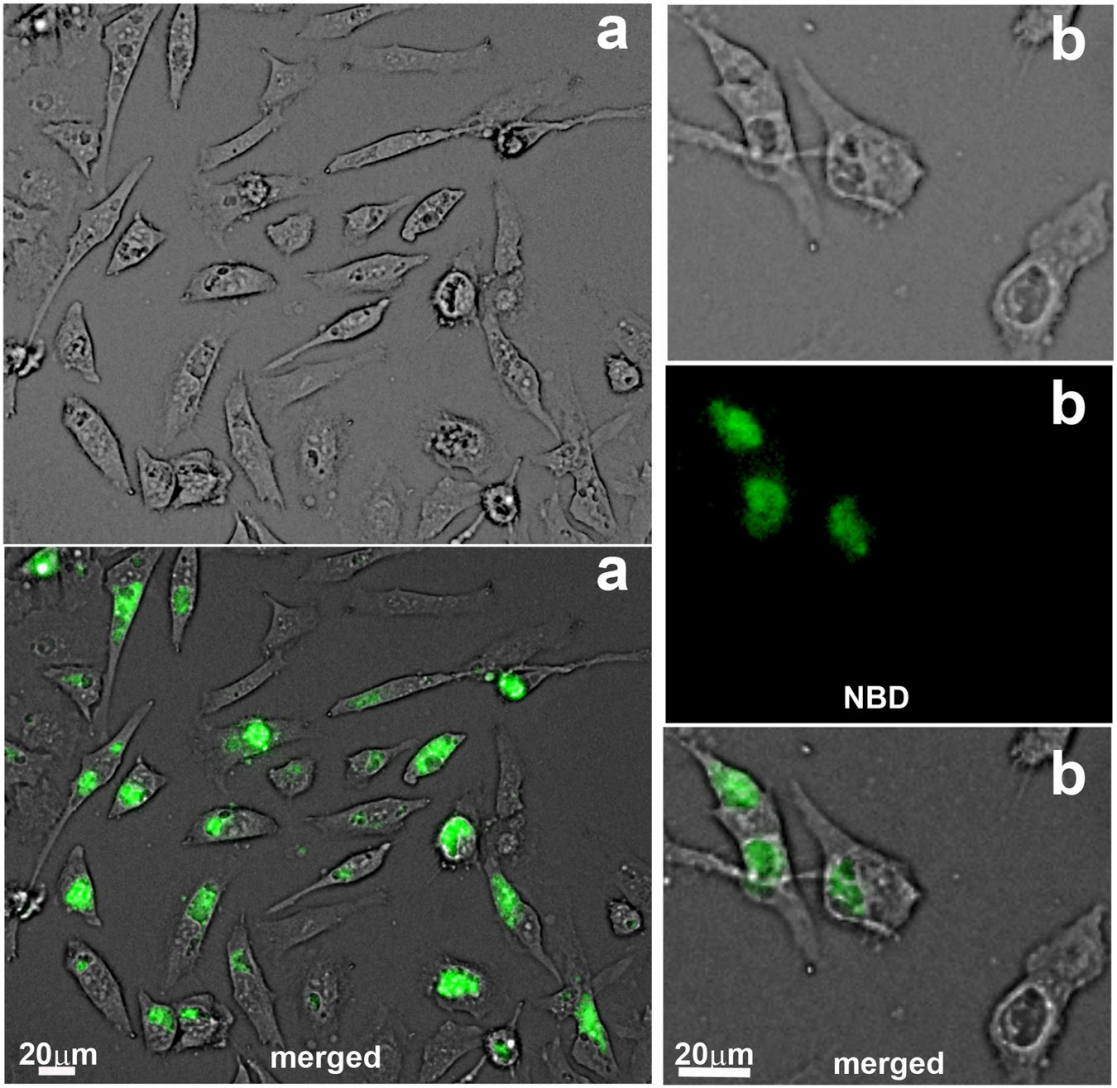
Labeling of *Chlamydia*-infected cells with 1-NBD-GPC. HeLa cells were grown in T-75 flasks and labeled with 5 µM 1-NBD-GPC (green) for 2 hours at 37 °C. The cells were washed and incubated in fresh medium for 2 hours before infection with *C*. *trachomatis* strain D. Live imaging was performed after 36 hours using a Keyence microscope equipped with a 20x objective (set a; bright-field and merged images with NBD fluorescence are shown). Note the presence of labeled and unlabeled cells. Cropped images (bright-field, NBD fluorescence, and merged images) showing 2 infected cells with labeled inclusions and one cell with an unlabeled inclusion are shown on the right (set b). Lipids were extracted from the treated cells and analyzed in the experiments presented in Fig. 7. Labeling experiments were performed twice and shown images were taken during the second experiment.

**Figure 7:**
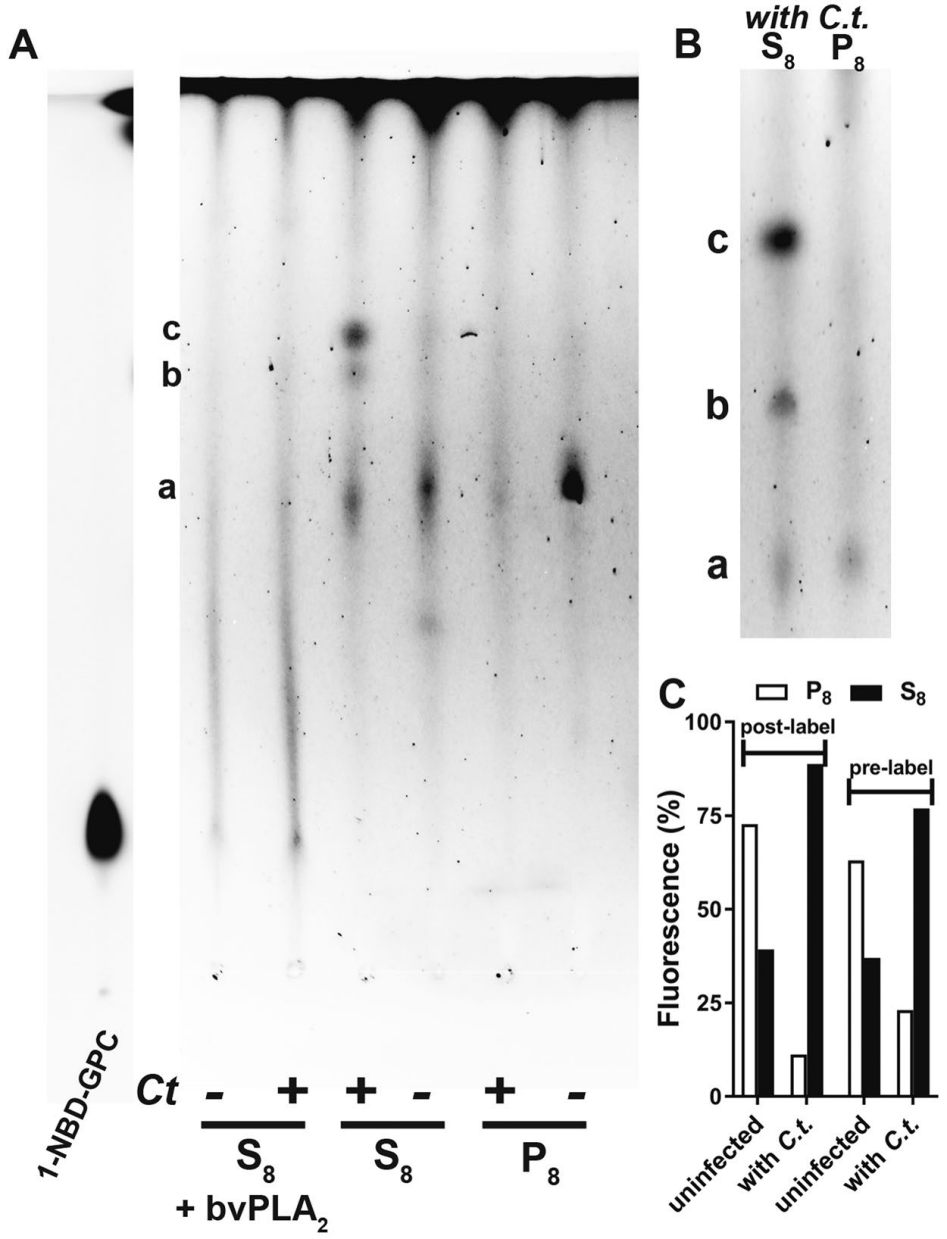
*In vivo* acylation of 1-NBD-GPC in *Chlamydia*-infected cells. Cells were grown and labeled as described in the legend of Fig. 6. Cells were either infected after labeling of the cells (pre-labeling conditions; images shown in Fig. 6) or were infected before addition of 1-NBD-GPC (post-labeling conditions; images shown in Figure S2). Cells were collected, washed, and lysed. The lysates were centrifuged at 8,000 g to generate pellet (P8) and supernatant (S8) fractions that represented the organelle and the cytosolic fractions enriched in EBs/RBs of infected cells, respectively. Lipids were extracted from the fractions obtained from pre-labeled cells (panel A) and post-labeled cells (panel B) and separated by TLC. Fluorescence quantification is shown in panel C. Labeled lipids obtained from pre-labeled cells were treated with bee venom PLA_2_ and analyzed (S8 + bvPLA_2_ in panel A). 1-NBD-GPC, which was used as a migration standard, was run on the TLC plate shown on panel A. The fluorescence of the lipids obtained from the samples was weaker than that of the standards, and the gamma intensity of the standard lane was adjusted to match that of the samples. For clarity, the image manipulation is indicated by presenting the standard lane as a separate section of the TLC plate on the left (NBD-LPC lane).

When fluorescent 1-NBD-GPC was added to the medium of infected cells, the host membranes and the inclusions were labeled (Figure S2A). EBs collected from the labeled cells developed fluorescent inclusions after infection of unlabeled cells (Figure S2B). The absence of labeling of the host cells indicated that the label was associated with the bacterial membranes. To further confirm the transfer of the 1-NBD-GPC molecule from the medium to the host membrane and subsequently to the bacteria, HeLa cells were labeled with 1-NBD-GPC prior to infection. The culture medium containing the unincorporated label was removed, and the cells were washed and then infected with *C*.*t*. After 24 hours, strong labeling of the inclusion was observed in the infected cells, and the label was no longer detectable in the host membranes (Fig. 6). A small fraction of the uninfected cells no longer displayed detectable labeling (Fig. 6, set a), and a few infected cells displayed inclusions with no labeling (Fig. 6, set b). The absence of the fluorescent dye from the culture medium leads to the presence of unlabeled newly divided cells. Infection of these cells may have resulted in the development of unlabeled inclusions. Infected cells obtained from cultures exposed to 1-NBD-GPC (post-labeled experiments; Figure S2) and cells labeled before infection (pre-labeled experiments; Fig. 6) were collected, and their fluorescent lipid content was analyzed by thin-layer chromatography. As shown in Fig. 7, several lipids (a, b and c) were detected, but 1-NBD-GPC was not recovered in any of the fractions. The presence of various fluorescent lipids in both pre-labeled (Fig. 7A) and post-labeled (Fig. 7B) infected cells provides strong evidence of lipid remodeling. In addition, the lipid profile of the fractions obtained from infected cells was not identical to the lipid profile of fractions obtained from uninfected cells (Fig. 7A). In particular, the signal intensity of the major lipid (c) detected in infected cells (fraction S8) was very low in uninfected cells, suggesting that it was formed in the *Chlamydia* inclusion. Furthermore, the major lipid (a) in the organelle fraction of uninfected cells (P8) was nearly undetectable in the organelle fraction of infected cells. Changes in the partitioning of the NBD fluorescence were quantified; the results confirmed a shift of the fluorescence to lipids in the S8 fraction of infected cells (Fig. 7C). This further supports the view that lipid pathways are re-routed to the bacteria in infected cells and that bacterial development alters host lipids. To confirm that fluorescently labeled lipids were obtained from the acylation of 1-NBD-GPC at the sn-2 position, the lipids (a, b, c) were digested with bee venom PLA_2_, and the products were analyzed (Fig. 7A). Following hydrolysis of the acyl group at the 2 position of the NBD-lipids, fluorescence was no longer associated with the lipids but instead co-migrated with 1-NBD-GPC. In addition, no increased fluorescence was observed in fatty acids (shown at the top of the plate). Thus, acylation of 1-NBD-GPC produced 1-NBD-2-acyl-PC. The presence of phospholipases, acyltransferases and fatty acid-activating enzymes of human and bacterial origin in the inclusions could account for the presence of the NBD acyl moiety of 1-NBD-GPC in various lipids (see Discussion). This remodeling of lipids led to products (a, b, c), which migrated differently from the synthetic standards NBD-PC, NBD-PE and NBD-PS. The specific identities of lipids a, b, and c were not established.

### Broad substrate range of Chlamydia acyltransferase CT775

**Figure 8:**
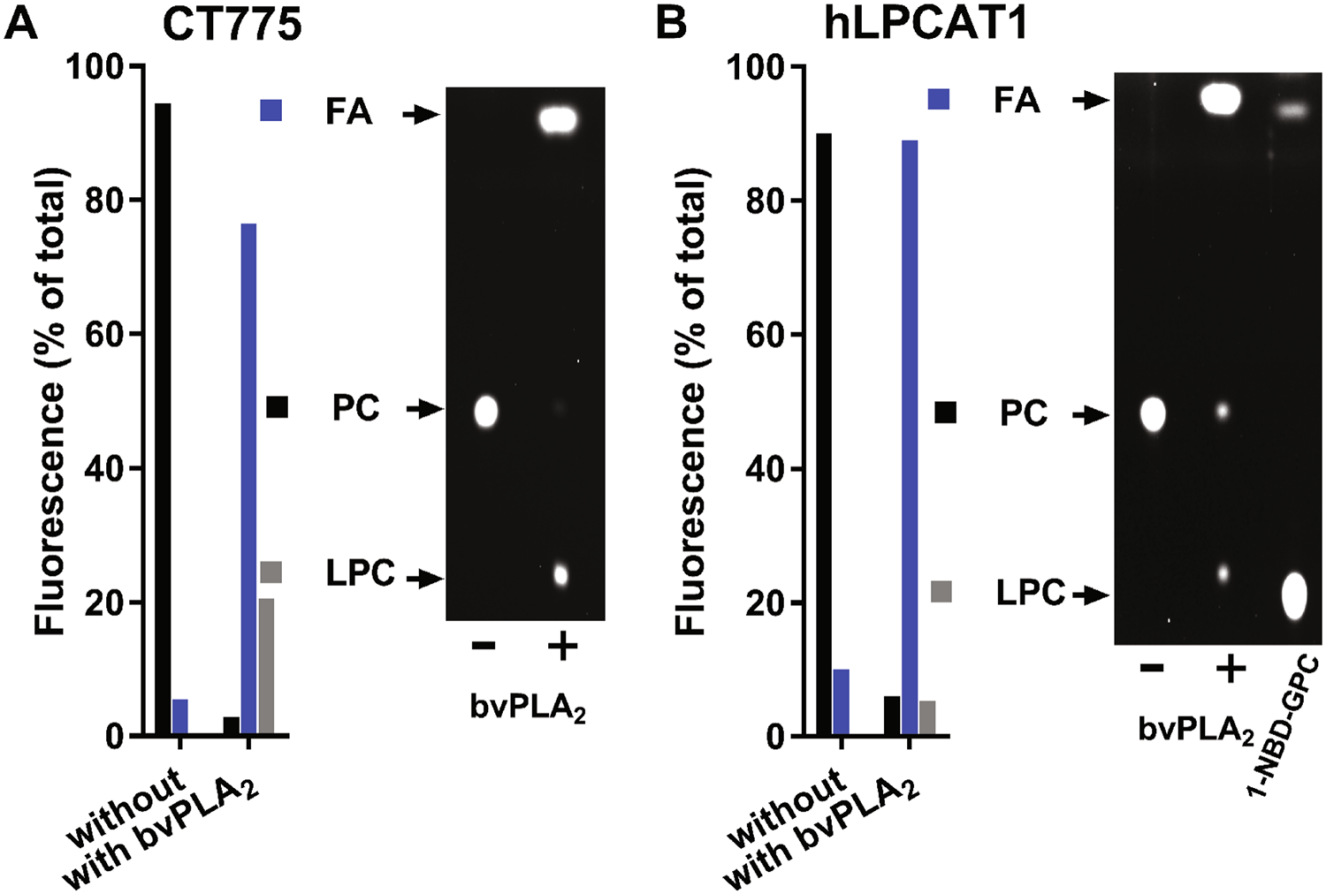
Acyl-CoA::1-acyl-GPC acyltransferase activity of CT775. CT775 was produced in *E*. *coli*, and activity in the membrane fraction was measured as described previously^30^. Reactions were performed with 1 µM NBD-C_16_-CoA in the presence of 10 µM synthetic 1-C_16_-sn-glycerol-3-P-choline (>90% 1-acyl-GPC isomer) at 30 °C. NBD-PC obtained after 30 minutes reaction with 40 µg of CT775 microsomes was extracted and separated by TLC. The NBD-PC spot was scraped from the plate and treated with bee venom PLA_2_ (bvPLA_2_). Lipids were extracted and separated by TLC, and the fluorescence in LPC (1- or 2-acyl-GPC), FA and unreacted PC was quantified. Experiments were performed with CT775 (panel A) and human LPCAT1 (panel B). NBD-LPC was used as a migration standard; it is shown in the right-hand lane of the TLC plate in panel B.

Several acyltransferases may have been responsible for the re-acylation of 1-NBD-GPC. In addition to the human LPCAT proteins, CT775 can acylate 1-acyl-GPC^30^. However, the activity of the acyltransferase enzyme CT775 (LpaT) appears to be controversial^31^ (see Discussion). In this study, the product of the reaction obtained in the presence of NBD-C_16_-CoA and synthetic 1-C_16_-sn-glycerol-3-P-choline (1-C_16_-GPC), which contained less than 10% of the 2-C_16_-GPC isomer (see Methods), was used to define the acyltransferase properties of CT775. Human LPCAT1, which catalyzes the transfer of acyl chains to the sn2 position^41,42^, was used as a control. Phospholipase A_2_ treatment of NBD-PC generated by either CT775 or LPCAT1 resulted in the release of the majority of fluorescence from the sn2 position of PC. Approximately 80 to 90% of the fluorescence was detected in the NBD-C_16_-OH fatty acid fraction (Fig. 8). This implied that the NBD-acyl group had been transferred by CT775 to the sn2 position of the 1-C_16_-GPC isomer and ruled out the possibility that CT775 is exclusively a 2-acyl-GPL acyltransferase^31^. However, approximately 20% of the fluorescence was associated with an 1-NBD-GPC product obtained from the NBD-PC generated by CT775 (Fig. 8A). This established that the NBD- C_16_ was also transferred to the sn1 position of the 2-C_16_-GPC isomer present in the reaction. Thus, our findings confirm the sn1 acyltransferase activity of CT775 reported by Yao *et al*. but establish that the enzyme can transfer acyl groups to both the sn1 and the sn2 positions of lysophospholipids. Moreover, like the *E*. *coli* PlsB acyltransferase, CT775 can also accept acyl-ACP and acyl-CoA as acyl donors.

### Acylation of 1-acyl-GPC from branched acyl-CoA by Chlamydia and human LPCAT enzymes

Lipids of *C*.*t*. contain odd-chain fatty acids (branched FA) that are synthesized exclusively by the pathogen^5,6^ (Figure S3). Two commercially available isomeric fatty acids, 11-methyl-lauric acid (MeC_12_-OH) and 17-methyl-stearic acid (MeC_18_-OH), were activated to CoA ester species by the human long-chain acyl-CoA synthetase hACSL6. The branched acyl-CoA products were purified, and the acyltransferase activity of the bacterial CT775 and human LPCAT1 enzymes was determined in their presence. hACSL6 was purified in its active form (Figure S3A). hACSL6 catalyzed a two-step reaction with an acyl-AMP intermediary product that accumulated in the absence of CoASH (Figure S3B). In the presence of both ATP and CoASH, acyl-CoA is formed from fatty acids. Competition of the formation of [^14^C]C_18:1_-CoA by MeC_18_-OH indicated that this long-chain odd fatty acid was as good a substrate for hACSL6 as C_18:1_-OH (Figure S3D). The shorter chain MeC_12_-OH also competed but not as efficiently as MeC_18_-OH.

**Figure 9:**
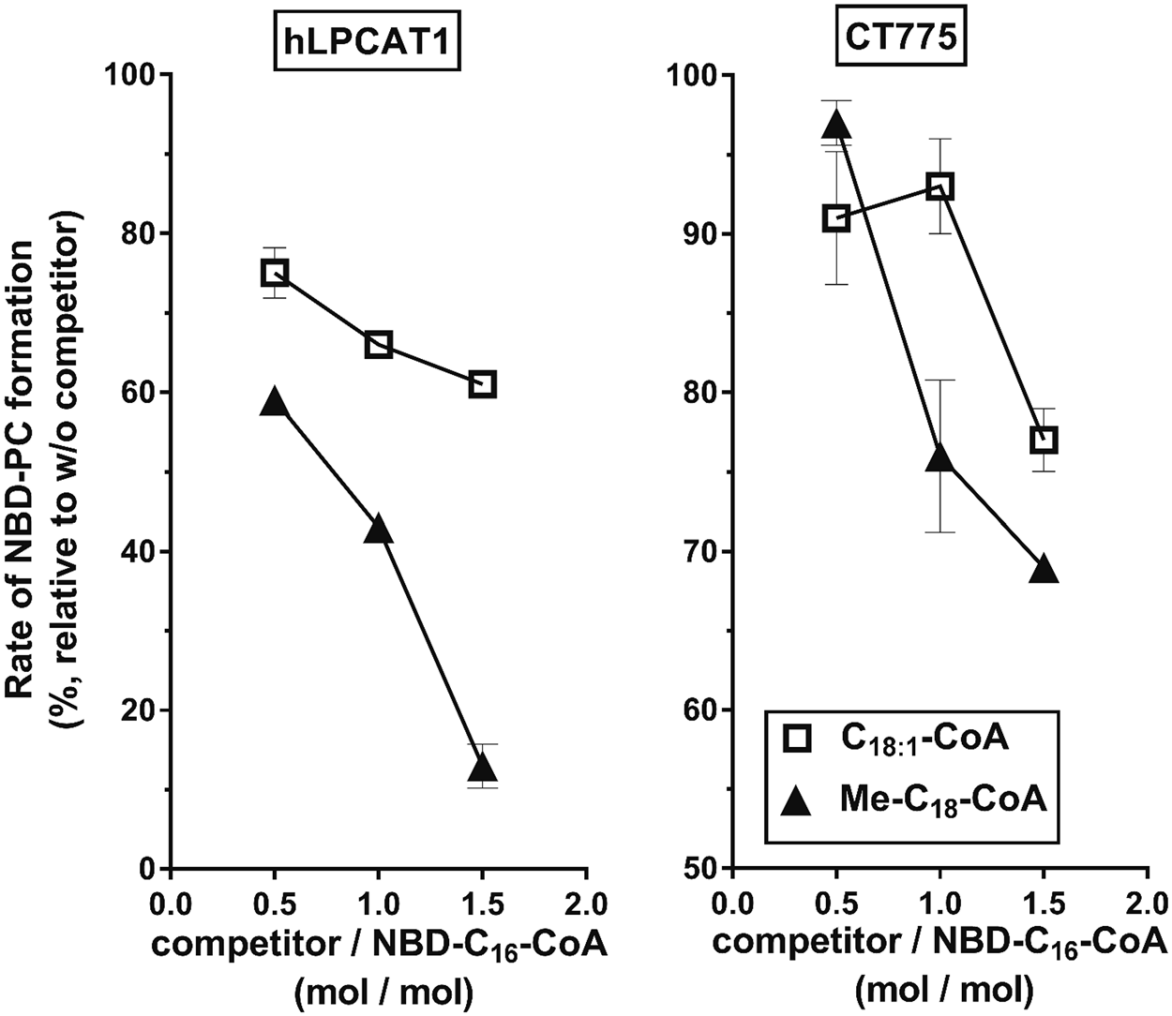
Odd-chain acyl-CoAs are substrates of hLPCAT1 and CT775. Human long-chain acyl-CoA synthetase 6 (hACSL6) was purified and used to produce CoA in the ester form from 17-methyl-stearic acid (MeC_18_-OH). MeC_18_-CoA was purified on a Lipidex-1000 column (see Methods), and its ability to compete with the acylation of 1-acyl-GPC from NBD-C_16_-CoA by human LPCAT1 (panel A) and CT775 (panel B) was determined. Measurements were performed with 0.5 µg of hLPCAT1 or CT775 microsomes in the presence of 20 µM 1-C_16_-GPC and 0.5 µM NBD-C_16_-CoA. The competitors C_18:1_-CoA and MeC_18_-CoA were added at concentrations of 0.25, 0.5 and 1.0 µM. For each condition, the rate of formation of NBD-PC was calculated from measurements of 3 samples taken between 0 and 6 min. The values obtained in the presence of the competitors are reported as percentages of the values obtained in their absence. Synthesis of odd-chain acyl-CoA was performed twice and were used in several independent competition experiments. The error bars indicate the standard deviation of 3 measurements.

The transfer of NBD-C_16_-CoA to 1-acyl-GPC by hLPCAT1 was strongly reduced in the presence of MeC_18_-CoA (Fig. 9). Although unsaturated C18 fatty acids are very abundant at the sn2 position of human PLs, MeC_18_-CoA was a stronger competitor than C_18:1_-CoA. For CT775, competition of NBD-C_16_-CoA was less efficient than for hLPCAT1, which might suggest a preference of the bacterial enzyme for the palmitic chain compared to the stearic chain. The *Chlamydia* acyl-ACP synthase AasC also has a substrate preference for C_16_ over C_18_^31^. As was the case for hLPCAT1, MeC_18_-CoA competed in the reaction, indicating that this branched acyl-CoA is a substrate for CT775 (Fig. 9). These findings strongly support the view that both the human and bacterial acyltransferases can acylate lysophospholipids with bacterial odd-chain fatty acids.

## Discussion

**Figure 10:**
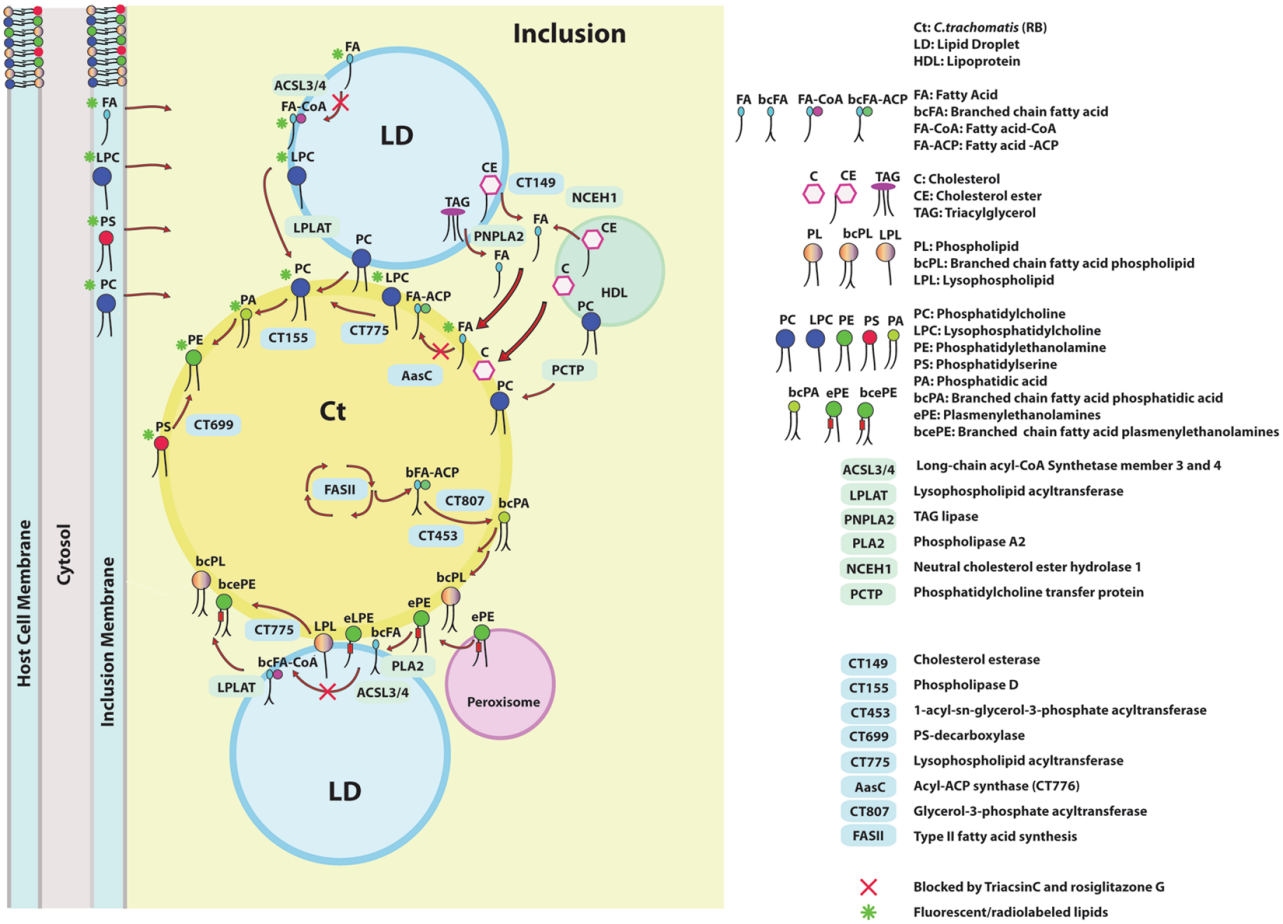
Model for FA and glycerophospholipids scavenging in *Chlamydia*-infected human cells. The inclusion contains pathways supporting the scavenging and remodeling of lipids obtained from the host membranes, organelles and vesicles. The model only presents enzymes and reactions that relate to the findings presented in this work, and that are described in detail in the Discussion section. Pathways essential for lipid *de novo* synthesis are not depicted and several pathways are simplified for clarity. Proposed mechanisms for the labeling of *Chlamydia* lipids by exogenously added fluorescent and radiolabeled lipids (PC, PS, LPC and FA) are highlighted with a green asterisk. Reactions that are sensitive to inhibition by the drugs triacsin C and rosiglitazone G are indicated with a red X.

The enzymes needed to capture and modify human fatty acids (FA) and lipids appear to be present in the intracellular inclusions formed by *C*. *trachomatis*. These enzymes catalyze reactions that include exogenous FA assimilation, lipid hydrolysis, re-acylation and head group remodeling (Fig. 10). The sources of FA are diverse. FA can be imported or released from lipids, cholesterol esters and TAG contained in vesicles (lipoproteins, LDs, and MVBs) and organelles (peroxisomes)^3,11,13,14,22,43,44^. Lipid hydrolysis can be catalyzed by several enzymes, including host cPLA_2_, which is recruited to the inclusion membrane and is activated during infection^45,46^. Human TAG lipase PNPLA_2_ is bound to LDs that are translocated into the inclusions in infected cells^20^. The bacterial cholesterol esterase CT149 and the host neutral cholesterol ester hydrolase NCEH1 can release FA from CE in the inclusion^17,47^. FA require activation to acyl-ACP or to acyl-CoA before their incorporation into lipids. Exogenous fatty acids can be activated to acyl-ACP by the *C*.*t*. acyl-ACP synthase AasC^31^ and to acyl-CoA by the host acyl-CoA synthetases hACSL3/ hACSL4^17,20,48^.

Bacterial lipids contain odd-chain fatty acids (branched-chain FA (BCFA)), and *C*.*t*. has its own set of enzymes that provide the activated BCFA-ACP precursor for the formation of these bacteria-specific lipids^31^. We show that purified hACSL6 can generate MeC_18_-CoA from MeC_18_-OH. If this reaction occurs in the inclusion, it would require that BCFA-OH be released from *de novo* synthetized BCFA-ACP to become a substrate of hACSL3-4. This putative pathway would be redundant for the formation of *C*.*t*. lipids produced *de novo* but could contribute to the modification of exogenous lipids that are present in the *C*.*t*. membrane (Fig. 10). One such reaction involves peroxisomes that are translocated into the inclusion and are in contact with RBs and LDs. Although the presence of intact peroxisomes in the inclusion has been disputed^49^, plasmenylethanolamines (PE plasmalogen) modified with bacterial C_15_ and C_17_ odd acyl chains at the sn-2 position have been identified in the membrane of *C*.*t* ^3^. The finding that the fluorescent precursor 1-NBD-GPC labels EBs indicates that acylation and incorporation of host lipids into *C*.*t*. membranes occurs in infected cells. The level of 1-acyl GPC in the membranes of infected cells decreased by 25%^6^. The level of the PC transfer protein PCTP is increased in infected cells^18^, and PCTP could participate in the translocation of PC to the inclusion via the CLA1 transporter^4^. PLA_2_ analysis of the labeled NBD-lipids found in our study confirmed that the label was used to remodel lipid at the sn2 position. Since LDs are in close contact with both peroxisomes and RBs, hACSL3/4 and one of the several 1-acyl-GPL acyltransferases bound to LDs might be responsible for the re-acylation of these host lipids. Human lysophospholipid acyltransferases (LPLAT) have been identified in LDs of infected cells (LPCAT1, LPCAT2, LPCAT4, ABHD5 (a soluble LPAAT)) and in the inclusion (LPCAT1, LPCAT3, MB0AT7 (LPIAT), GAPT3)^17,20^. The activity of the acyltransferase CT775 (LpaT) appears to be controversial. Yao and colleagues proposed that CT775 exclusively uses acyl-ACP as donor and that it transfers the acyl group specifically to the 1 position of 2-acyl-glycerophospholipids^31^. In our studies, we have now confirmed the transfer of straight-chain acyl from acyl-CoA to the 2 position of 1-acyl-GPC and ruled out the conclusions of Yao *et al*. regarding the narrow substrate range of the enzyme. These findings are not contradictory but establish that CT775 is an acyltransferase with broad substrate specificity. We show that human LPCAT1 and CT775 accept iso Me-C_18_-CoA as an acyl donor. Thus, straight-chain FAs scavenged from the host as well as *de novo* synthesized odd-chain FAs could be incorporated into *C*.*t*. lipids via re-acylation by the host and bacterial acyltransferases.

In addition to these re-acylation processes, the bacterium can modify PL head groups. Lipidomic analysis shows a 70% decrease in PS and a 60% increase in PE in infected cells^6^. Increased levels of PE, the most abundant lipid in the *C*.*t*. membrane, can be attributed to both *de novo* bacterial synthesis and remodeling of PS by the action of the PS-decarboxylase CT699. Although *C*.*t*. synthesizes PS *de novo*, the observed decrease in the PS level in infected cells indicates the importance of scavenging and remodeling of the host PS. In addition to the modification of PS to PE, head groups can also be replaced by the action of phospholipase D (PLD), and several putative PLD enzymes are produced by the bacterium. PLD CT155 was localized to the membrane of RBs associated with the inclusion membrane and may participate in the remodeling of imported lipids^50^. Modification of host PC with odd-chain fatty acids was detected in one study^5^ but was not confirmed in other studies^4,6^. Wylie *et al*. reported that labeling of PC by the odd-chain fatty acid precursor^14^C-isoleucine was highest early in infection (50% of that of PE) but decreased to approximately 1/5 that of PE at 48 hours post-infection^5^, which was the time at which the samples analyzed by mass spectrometry were collected in the other two studies^4,6^. Analysis at an earlier time point might have led to the identification of modified host-derived PC molecules in the *C*.*t*. membrane. The presence of large amounts of straight-chain host PC and odd-chain bacterial PE in the analyzed samples may have prevented identification of the rarer PC molecular species modified with odd chains. It is possible that PC species containing iso and anteiso fatty acids were among the several molecular mass peaks that were detected but were not assigned a molecular species identification^6^.

The effects of rosiglitazone G and triacsin C on ACSL activity have been determined *in vitro* either with purified proteins or in cell lysates that were exposed to the drug during the reaction^34,51^. The defective growth of *C*.*t*. in cells treated with those drugs has been attributed to inhibition of the activity of ACSL family members^36,37^. However, the interpretation of *in vivo* experiments of this type is hampered by the ambiguity of the location of the various ACSL forms and the fact that the degree of inhibition of each of the five ACSL enzymes by these drugs has not been determined *in vivo*. Several reports have indicated the presence of ACSL3 and ACSL4 in the inclusion using immunodetection and proteomic analysis, and the association of these enzymes with LDs was shown to increase significantly in infected cells^17,20,48^. However, ACSL1, ACSL5 and ACSL6 were not detected. Using an affinity-purified antibody and a GFP-tagged recombinant form of hACSL6, we have shown that hACSL6 was not detected in the inclusion^48^. In the study of Recuero-Checa *et al*., immunodetection of hACSL5 and hACSL6 was performed using antibodies of undefined specificity. In that study, cells in which hACSL1 expression had been down-regulated were stained with a signal intensity similar to that observed in control positive cells^37^. These antibodies may have cross-reacted with other hACSL forms and could have led to their apparent detection in the inclusion^37^.

In this work, we showed that rosiglitazone G and triacsin C inhibited FA incorporation by *C*.*t*. acyl-ACP synthase AasC. In contrast to *C*.*t*. AasC, the *E*. *coli* enzyme, which is not sensitive to the drugs, does not liberate acyl-ACP; instead, it channels it directly to the acyltransferase domain for transfer to 2-acyl-GPE^33,38^. In *C*.*t*., the uncoupling of the acyl-ACP synthase and the acyltransferase reaction, which can be performed with acyl-CoA, could confer sensitivity to drugs that are inhibitory to acyl-CoA synthetases. Other acyl-ACP synthases are inhibited by triacsin C^52^. Other pathways that provide lipids to the bacterium need to be taken into consideration when treating cells with these drugs. Decreasing the size of the acyl-CoA pool in inhibitor-treated infected cells will result in decreased esterification of cholesterol by ACAT, which appears to be essential to the bacterium^53^. Lipid droplets (LD) are translocated and consumed in the inclusion and are essential for *C*.*t*. development^20^. We found that FA incorporation into TAG, which forms the core of LDs, was greatly decreased in inhibitor-treated cells. Since reduction of LD formation affects *C*.*t*., it cannot be concluded that the inhibitory effect of the drugs did not involve LD formation, as suggested^37^. Rosiglitazone G and triacsin C treatment were performed in the absence of oleic acid (an inducer of LD formation) and in the presence of gentamicin, vancomycin and nystatin, which can alter the cholesterol composition of membranes^54^. The lack of interaction of these drugs with rosiglitazone G or triacsin C has not been established, and their presence in the medium might have altered the lipid composition and metabolism of the infected cells. This could account for the unexplained finding that the ACSL activity measured in the absence of the two inhibitors was lower in lysates of cells that were grown in the presence of the inhibitors than in lysates of control cells.

In conclusion, we provide evidence that *C*. *trachomatis* possesses an extended capability to generate its own lipids. The salvage pathway of exogenous fatty acids by bacterial AasC/CT775 and host ACSL3-4/LPLAT might represent a redundant and complimentary dual system that can adapt to changes in the host environment and metabolism to prevent disruption in the development of the membrane of the bacterium.

## Materials and Methods

### Materials

Radiolabeled [1-^14^C]C_16_-OH and [1-^14^C]C_18:1_-CoA were purchased from PerkinElmer (Hopkinton, MA, USA). NBD-PS (1-palmitoyl-2-(6-[(7-nitro-2-1,3-benzoxadiazol-4-yl)amino]hexanoyl)-sn-glycero-3-phosphoserine), 1-NBD-GPC (1-(12-[(7-nitro-2-1,3-benzoxadiazol-4-yl)amino] dodecanoyl)-2-hydroxy-sn-glycero-3-phosphocholine), 1-acyl-GPC (1-palmitoyl-2-hydroxy-sn-glycero-3-phosphocholine, >90% pure) and NBD-C_16:0_-CoA (N-[(7-nitro-2-1,3-benzoxadiazol-4-yl)-methyl]amino palmitoyl Coenzyme A) were from Avanti Polar Lipids, Inc. (Alabaster, AL, U.S.A). C_1_-BODIPY 500/510 C_12_ (4,4-Difluoro-5-Methyl-4-Bora-3a,4a-Diaza-s-Indacene-3-Dodecanoic Acid) was from ThermoFisher Scientific. TLC silica plates were obtained from Analtech. Inc. (Newark, DE, U.S.A). Honey bee venom Phospholipase A_2_, 11-Methyl-lauric acid (MeC_12_-OH), 17-Methyl-stearic acid (MeC_18_-OH), C_18:1_-OA, C_18:1_-CoA, triacsin C and Rosiglitazone G were from Sigma-Aldrich.

### DNA manipulation and protein expression

All PCR cloning reactions were performed with High-Fidelity Expand Taq DNA polymerase (Roche Applied Science). All amplicons were cloned with the Zero-Blunt PCR cloning kit (Life Technologies), and their identity was verified by sequencing. Full-length *Chlamydia trachomatis serovar D CT699* (PS-decarboxylase) and *CT776* (AasC) genes were cloned into the pET28a vector (Novagen). Cloning of *hLPCAT1* and *CT775* was reported previously^30,41^. Proteins were produced in BL21DE3 cells as previously described^42^. Expression was induced by addition of IPTG at a final concentration of 0.5 mM and cells were harvested after 2 to 3 hours of growth at 37 °C. For hLPCAT1 and CT775, membrane factions were prepared as previously described^42^.

### hACSL6 purification

Cloning and expression of recombinant hexahistidine fusion of human ACSL6 isoform2 has been previously described^55^. Cells were lysed in 20 mM sodium phosphate buffer pH 7.4, 500 mM NaCl, 10 mM imidazole, 10 mM MgCl_2_, 2 mM DTT, 1 mM PMSF, and 1% CHAPS and passed through a Ni-NTA column. Partially purified hACSL6-iso2 was snap-freeze and kept at −80 °C until use.

### Branched-chain acyl-CoA synthesis, acyl-CoA synthetase assays and LPCAT competition

Acyl-CoA synthetase assays were performed with [^14^C]C_18:1_-OH in presence of ATP and CoASH as described previously^55^. Reactions were performed in 150 µl with 1 µM^14^C]C_18:1_-OH, 10 µM competitors and 0.15 µM hACSL6-iso2 at 30 °C. Production of larger amounts of MeC_12_-CoA and MeC_18_-CoA (used in competition experiments) was performed in 600 µl with 10 µM of 11-Methyl-lauric acid (MeC_12_-OH) or 17-Methyl-stearic acid (MeC_18_-OH) and 0.06 µM hACSL6-iso2. Reactions were incubated at 30 °C for 30 minutes. Unreacted fatty acids were separated from acyl-CoA products by passage of the mixture through a Lipidex-1000 column at 4 °C. A Rose-Oaklander extraction^56^ was preformed and the isolated acyl-CoAs were dried and suspended at a calculated concentration of 3 µM in 150 µl 20 mM Tris-HCl pH 7.4 with 0.08% Tween-20. Competition experiments were performed in 200 µl at 30 °C with 0.5 µg of enzyme (CT775 or hLPCAT1) in the presence of 0.5 µM NBD-C_16_-CoA and 0.25-0.5-1 µM of the competitors, and 20 µM 1-acyl-GPC. After 2, 4 and 6 minutes incubation, 50 µl of the reaction was removed and transferred into 190 µl CHCl_3_/MeOH (1/2; v/v) and lipids were extracted according to the Bligh and Dyer method^57^. Lipids were separated by thin-layer chromatography on silica plates developed in the Skipski system made of chloroform/methanol/acetic acid/0.9% NaCl (100:50:16:5, v/v)^58^. Fluorescent spots were visualized with a FluoChem camera (Bio-Rad) and identified using 1-NBD-GPC and NBD-PC as migration standards. Quantification was performed with Quantity One software (Bio-Rad).

### PS-decarboxylase activity assays

Activity of CT699 was measured in *E*. *coli* BL21DE3 lysate made in Tris-HCl 0.1 M pH 7.4 supplemented with 10% glycerol and was compared to values of the activity obtained in cells transformed with the pET28a vector. Decarboxylation of NBD-PS to a fluorescent PE molecule was performed in 100 µl Tris-HCl 20 mM pH 7.4 with 1 µg of proteins lysate from 0 to 6 minutes at 37 °C. Reactions were stopped by lipid extraction with the Bligh and Dyer method. Products were separated by TLC as described above. Fluorescent spots were identified using NBD-PS and NBD-PE as migration standards.

### Acylation of 1-acyl-GPC and PLA_2_ treatment

To assess the transfer of the acyl chain from acyl-CoA to the sn-2 position of 1-acyl-GPC by hLPCAT1 and CT775 proteins, fluorescent PC was obtained following incorporation of NBD-C_16_-CoA into synthetic 1- C_16_-GPC (>90% 1-palmitoyl-2-hydroxy-sn-glycero-3-phosphocholine and 10%< of 1-hydroxy-2- palmitoyl-sn-glycero-3-phosphocholine). The products were subsequently treated with bee venom PLA_2_. Acyltransferease reactions were performed with 10 µM 1-C_16_-GPC (90% > pure) and 1 µM NBD-C_16_-CoA in 600 µl of 20 mM Tris-HCl pH 7.4, 5 mM β-mercaptoethanol, 5 mM EDTA and with 0.8 mg/ml Tween-20. Reactions were initiated by the addition of 40 µg proteins and terminated after 30 minutes incubation at 30 °C by extraction of lipids with the Bligh and Dyer method. Following isolation and extraction, NBD-PC was digested with 10 units of bee venom PLA_2_ (Sigma) in 100 µl of 25 mM Tris-HCl pH 8, 10 mM CaCl_2_, 100 mM KCl with 0.3 mM triton X-100 at 30 °C for 30 minutes. Reactions were stopped and extracted with the Bligh and Dyer method. Lipids were separated on silica plates and fluorescent spots were visualized with a FluoChem camera (Bio-Rad) and identified using NBD-PC, NBD-lysoPC and NBD-acyl as migration standards. Quantification was performed with Quantity One software (Bio-Rad).

### Triacsin C and rosiglitazone G treatment in *E. coli*

*E*. *coli* strain BL21(DE3) transformed with the expression plasmid pET28::CT776 (AasC) was grown in LB rich medium at 37 °C to an OD_600_ of about 0.4 and expression was induced for 1 hour with 0.5 mM IPTG. Un-induced cells were grown to an OD_600_ of about 0.7. Cells were harvested and were lysed with a FrenchPress in 50 mM Tris-HCl pH 7.4, 100 mM NaCl, 5 mM DTT and 10% glycerol. Reactions were performed in 450 µl of 50 mM Tris-HCl pH 7.4, 20 mM MgCl_2_, 3 mM DTT, 10 mM ATP and 5 µM [^14^C]C_16_-OH, which was suspended in 20 µl 0.55 mM Triton-X100. When mentioned, CoASH was added at 0.5 mM and 1-acyl-GPC at 20 µM. Reactions were initiated by the addition of 50 µg of protein extract and incubated at 30 °C. At the indicated times, 100 µl of the reaction was removed and transferred into 375 µl of CHCl_3_/MeOH (1/2; v/v) and lipids were extracted with the Bligh and Dyer method. Stock solution of triacsin C and rosiglitazone G were made in 98% ethanol at a 100x concentration and 4.5 µl was added before initiating the reaction. Control reactions were performed in the presence of 4.5 µl of 98% ethanol. Lipids were separated on TLC plates as described above. TLC plates were air-dried and exposed to a PhosphoImager screen (Storm 840, Molecular Dynamics). Quantification of the rate of formation of [^14^C]PLs was performed with ImageQuant software after subtraction of the plate background.

### Cell culture, infection and labeling

HeLa229 cells were maintained in minimal essential medium (MEM alpha, Invitrogen, Carlsbad, CA) containing 10% fetal bovine serum and 2 mM glutamine. Cells were infected with *Chlamydia trachomatis serovar D* as described previously^30^. Live imaging was performed with cells grown in T75 flasks with a Keyence microscope equipped with a 20x objective. Imaging of NBD-PS incorporation was performed with cells grown and infected on coverslips for 24 hours in 12-well plates. Fixed cells were stained with the DNA dye Hoechst 33258 as previously described^30^.

### Triacsin C and rosiglitazone G treatment in *C.t*.-infected HeLa cells

To determine the effect of the drugs on the rate of incorporation of fatty acids into lipids, infected and un-infected cells were labeled with a fluorescent fatty acid analog (C_1_-BODIPY 500/510 C_12_). Subsequently, the drugs were added and cells were labeled with [^14^C]C_16_-OH. The amount of fluorescent lipids was used as an internal reference to calculate the inhibition of incorporation of [^14^C]C_16_-OH in the presence of the drugs compared to incubations without the drugs. To minimize changes in the relative amounts of *C*.*t*. and host lipids in the untreated and treated samples, drug treatments were limited to two hours. Un-infected cells were grown to about 80% confluence and were treated as indicated for the infected cells. When inclusions in infected cells were large enough to occupy most of the cytosolic space of the cells, usually after 30 hours (as seen in Figure S2), 5 µM C_1_-BODIPY 500/510 C_12_ was added to the medium and cells were incubated for 2 hours. Medium was removed, cells were washed twice with 0.05% BSA in PBS, twice with PBS and fresh medium was added. Triacsin C was added at a concentration of 10 µM and rosiglitazone G at 100 µM. An identical volume of ethanol was added to the untreated cells. Cells were incubated for 1 hour and subsequently, 10 µM of [^14^C]C_16_-OH complexed with BSA was added to the medium, and further incubated for 1 hour at 37 °C. A 1.2 mM solution of [^14^C]C_16_-OH was made with 0.02% BSA and 50 µl of that mixture was added to 6 ml of medium. The medium was removed, cells were washed twice with BSA 0.05% made in PBS, twice with PBS, and twice with water. Cells were scraped in 4 ml water and un-infected cells were collected by centrifugation at 3,000 g for 10 minutes. For infected cells, centrifugation was performed at 14,000 g for 30 minutes. The cells and EBs/RBs pellet were saved and the supernatant was centrifuged again. The second pellet was pooled with the first. Lipids were extracted with the Rose-Oaklander method. The precipitated proteins were solubilized in 2% SDS and insoluble particles were removed by centrifugation at 14,000 g for 15 minutes. Proteins were then analyzed by western-blotting for *C*.*t*. HSP60 and hGAPDH with mouse monoclonal antibodies. The protein concentration was determined using the Bradford reagent with BSA as standard. Phospholipid concentrations were assessed by the Rouser method^59^. An equivalent amount of lipids (600 ng) was spotted on each lane of TLC silica plates. Two set of plates were run. Phospholipids were separated in a system of chloroform/methanol/acetic acid/0.9% NaCl (100:50:16:5, v/v)^58^, and TAG in hexane/diethylether/acetic acid (105:43:3)^34^. Fluorescent lipids were visualized with a FluoChem camera, and the radio-labeled lipids were then detected after exposure of the plate to a PhosphoImager screen. Quantification of the amount of fluorescent and radio-labeled lipids in each of the extract obtained from un-infected and infected cells, in the presence or absence of drugs, was performed with ImageQuant and QuantityOne software.

### Incorporation of NBD-labeled lipids by HeLa infected cells and bee venom PLA_2_ treatment

NBD-PS was added to the medium at a concentration of 1 µM and infected cells were incubated for 1 hour at 37 °C. Medium was removed, cells were washed twice with 1% BSA in PBS and twice with PBS. Fresh medium was added and cells were incubated for another 3 hours. 1-NBD-GPC labeling was performed with *C*.*t*.*-*infected HeLa cells (post-labeling condition) or with cells prior to infection (pre-labeling conditions) as follows. HeLa cells infected with *C*.*t*. for 16 hours were incubated with 5 µM 1-NBD-GPC for 1 hour. Cells were washed and incubated with fresh medium for 5.5 hours, as described above. Live imaging was performed during the first hour of the incubation period after removal of the dye (Figure S2 panel A). In one set of experiments, cells were then scraped, collected, and lysed with a Dounce homogenizer to obtain a crude preparation of infectious *C*.*t*. elementary bodies (EB), which was used to infect a new flask of un-labelled HeLa cells. Live imaging was performed after 14 hour to monitor the presence of fluorescent *C*.*t*. inclusions (Figure S2 panel B). In another set of experiments (Fig. 7), the cells were collected for lipid analysis as described below. HeLa cells grown to a confluence of about 70% were first incubated with 1-NBD-GPC for 2 hours, washed and incubated in fresh medium for another 2 hours. Pre-labeled HeLa cells were then infected with *C*.*t*. and grown for 36 hours. Live imaging of cells was performed (Fig. 6). Then, the cells were scraped in water and lysed with Dounce homogenizer. The lysate was centrifuged at 8,000 g to yield P8, which was washed once in water. The supernatant, S8, represented the lysate fraction containing the EBs/RBs from infected cells. Lipid extraction was performed with the Rose-Oaklander method. Lipids were separated on TLC plates and fluorescent PL were scraped, collected and treated with bee venom PLA_2_ as described above.

## Electronic supplementary material

Supplementary Figures
