# Supplementary material for: Phosphatidylserine decarboxylase CT699, lysophospholipid acyltransferase CT775, and acyl-ACP synthase CT776 provide membrane lipid diversity to Chlamydia trachomatis
Source: Sci Rep. 2017 Nov 17;7:15767. doi: 10.1038/s41598-017-16116-8 (PMC5693948; doi:10.1038/s41598-017-16116-8)

Supplementary Info File

Phosphatidylserine decarboxylase CT699, lysophospholipid acyltransferase CT775, and acyl-ACP synthase CT776 provide membrane lipid diversity to *Chlamydia trachomatis*

Eric Soupene, and Frans A. Kuypers

Children's Hospital Oakland Research Institute, Oakland, CA, USA

SUPPLEMENTARY FIGURES LEGEND

Figure S1: Measurements of *C.t.* acyl-ACP synthase AasC activity in *E.coli*

C.t. AasC (CT776) was expressed in *E.coli* and incorporation of 5 µM ^{14}C-C$_{16}$-OH was determined in lysate. Panel **A**; Reactions were performed at 30°C and 4 samples were taken from 0 to 8 min as indicated on top of the TLC plate. Reactions were performed with or without ATP and CoASH, as indicated at the bottom of the TLC plate. Panel **B**. The product of the reaction obtained at 8 minutes in presence of ATP and ^{14}C-C$_{16}$-OH (lane e.c. + ATP + C$_{16}$-OH) was run with the standards ^{14}C-PA (lane 2), ^{14}C-PC (lane 3) and ^{14}C-PE (lane 4). Panel **C**. Reactions were performed as in panel A in presence of 20 µM of 1-acyl-GPC. Note the presence of the new product PC in addition of PE. Panel **D**. Reactions were performed as in panel C in presence of ^{14}C-C$_{16}$-CoA instead of ^{14}C-C$_{16}$-OH. Products of the reaction obtained with ^{14}C-C$_{16}$-OH at 8 minutes was included as control (left lane).

Figure S2: Labeling of *Chlamydia*-infected cells with 1-NBD-GPC

HeLa cells were grown in T75 flasks and infected with *C.t.* for 30 hours. Infected cells were labeled with 5 µM 1-NBD-GPC (green) for 2 hours at 37°C. The cells were washed, and incubated in fresh medium for 5.5 hours. Live imaging was performed during the first hour with a Keyence microscope equipped with a 20x objective (panel A). Set a and Set b show the cropped images of two different infected cells with the NBD label detected in the plasma membrane of the host cells and in the *C.t.* inclusion. The bright-field and merged fluorescence images are shown. Panel **B**. EBs collected from the culture shown in panel A were collected and used to infect a flask of unlabeled HeLa cells. Set a, b, c and d show NBD-labeled small inclusions in 4 different infected cells. Note the absence of labeling of the host cell membranes.

Figure S3: Purification and activity of hACSL6

Human long-chain acyl-CoA synthetase member 6 (hACSL6) was purified and activity was tested in two of the purified factions (panel **A**) in presence of ^{14}C-$C_{18:1}$-OH, ATP and CoASH. Product of the reactions obtained in absence or presence of ATP and CoASH, as indicated, were analyzed by thin-layer chromatography (panel **B**). The positions of the unreacted substrate, of the intermediary product acyl-AMP and, of the product acyl-CoA are indicated on the right of the plate. Panel **C** shows a cartoon representation of odd chain fatty acids in the iso and anteiso conformation compared to a straight-chain FA. Panel **D**. Formation of ^{14}C-$C_{18:1}$-CoA from ^{14}C-$C_{18:1}$-OH by purified hACSL6 was measured in presence of 11-Methyl-lauric acid (MeC_{12}-OH), 17-Methyl-stearic acid (MeC_{18}-OH) and, of $C_{18:1}$-OH. Inset: Rates of formation of ^{14}C-$C_{18:1}$-CoA in presence of the competitors were calculated relative to the value obtained in their absence and are presented as percent.

SUPPLEMENTARY FIGURE 1

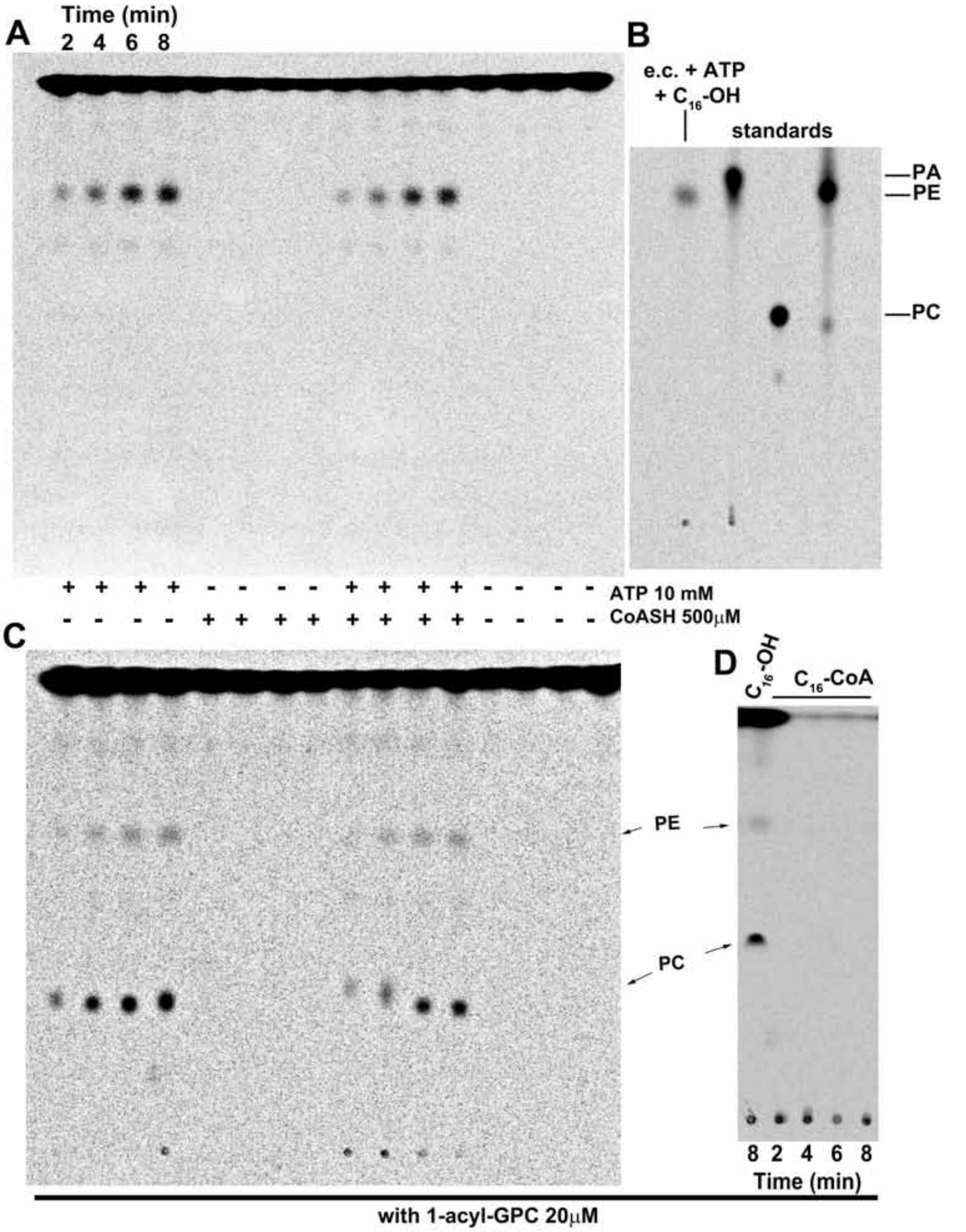

SUPPLEMENTARY FIGURE 2

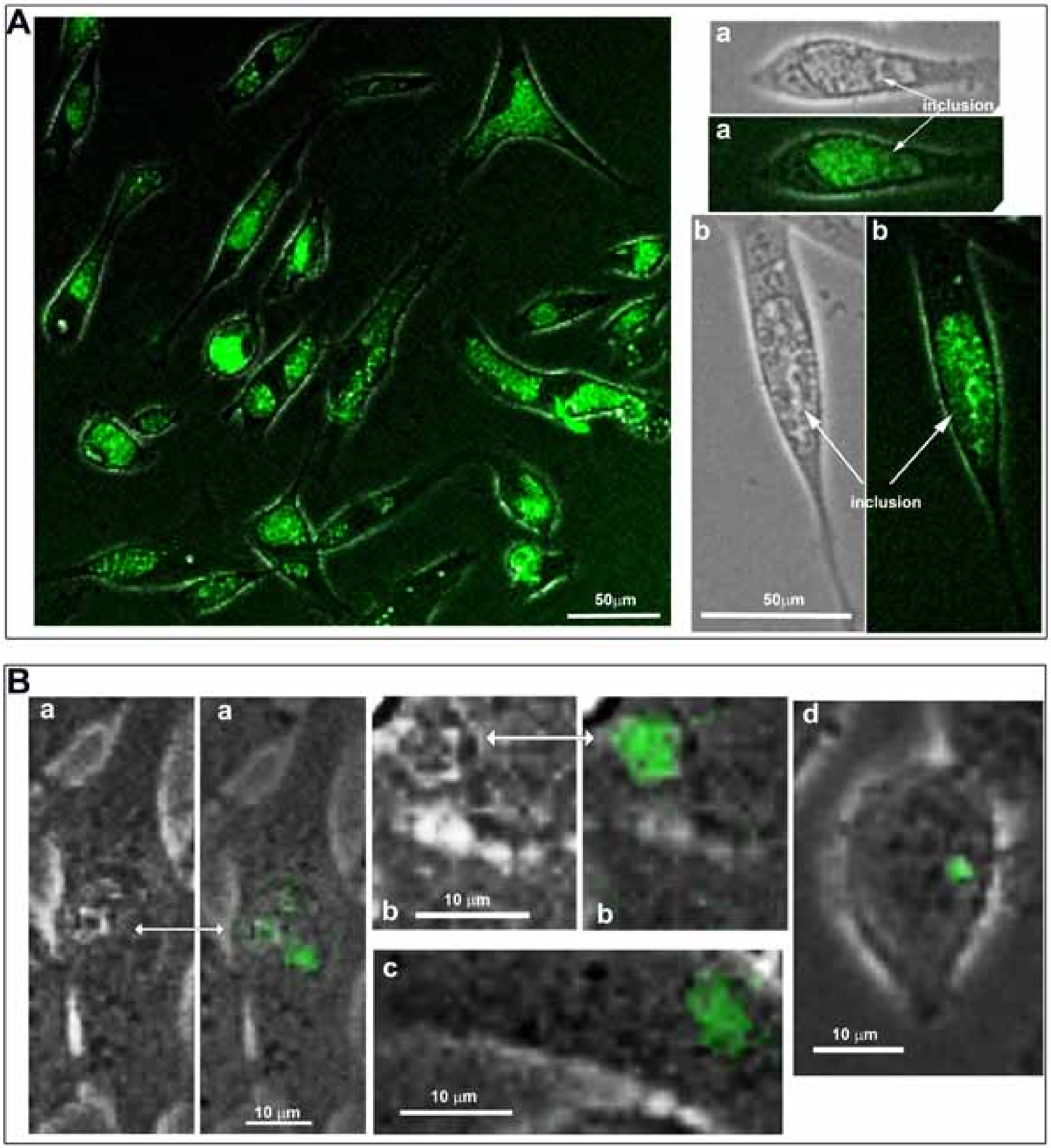

SUPPLEMENTARY FIGURE 3

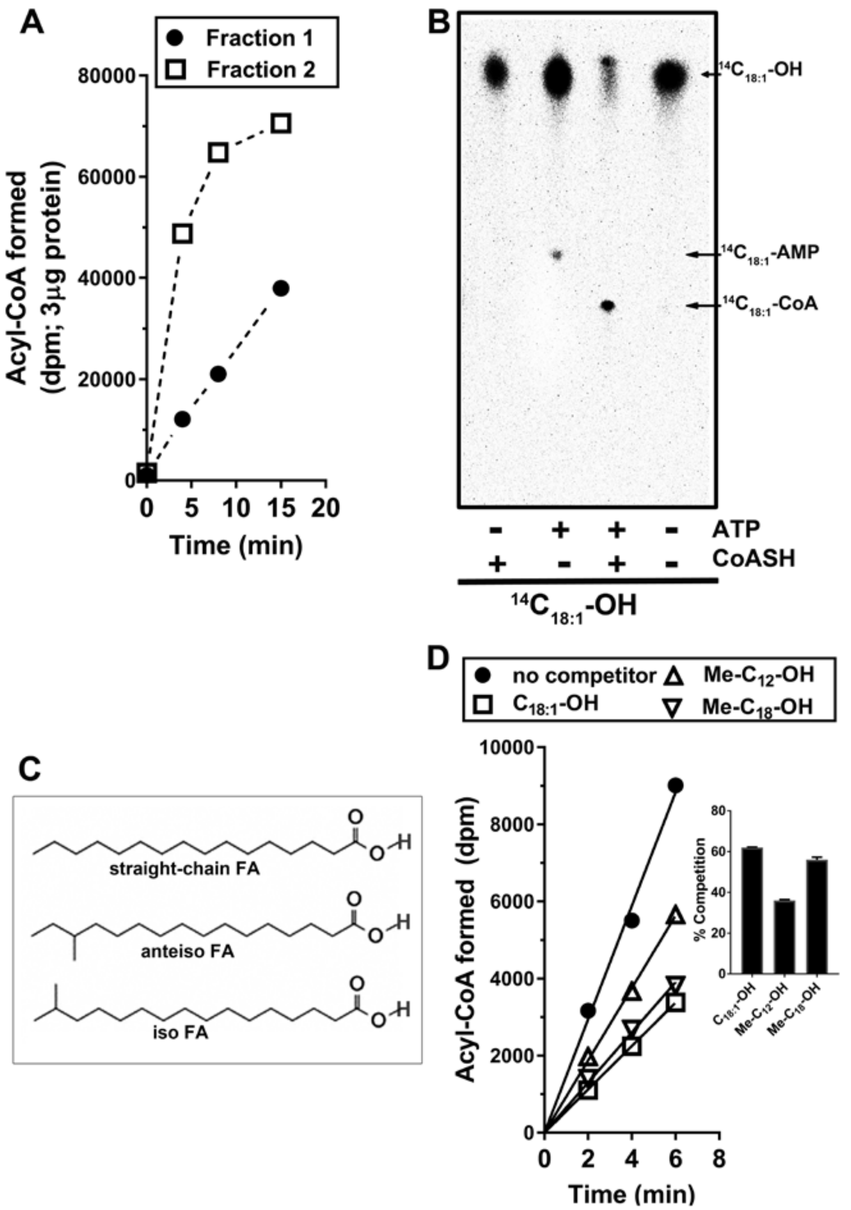

Supplement: Supplementary file 1 — Supplementary Figures [file 41598_2017_16116_MOESM1_ESM.pdf]
